# Formation, Structural Characterization, and Functional Properties of Corn Starch/Zeaxanthin Composites

**DOI:** 10.3390/foods12102076

**Published:** 2023-05-22

**Authors:** Songnan Li, Duo Feng, Enpeng Li, Robert G. Gilbert

**Affiliations:** 1 Joint International Research Laboratory of Agriculture and Agri-Product Safety of the Ministry of Education of China, Institutes of Agricultural Science and Technology Development, Yangzhou University, Yangzhou 225009, China; lsnyz2020@yzu.edu.cn (S.L.); fd_yzu@163.com (D.F.);; 2 Laboratory of Crop Genomics and Molecular Breeding/Key Laboratory of Plant Functional Genomics of the Ministry of Education/Jiangsu Key Laboratory of Crop Genetics and Physiology, Agricultural College of Yangzhou University, Yangzhou 225009, China; 3 Jiangsu Co-Innovation Center for Modern Production Technology of Grain Crops, Yangzhou University, Yangzhou 225009, China; 4 Centre for Nutrition and Food Sciences, Queensland Alliance for Agriculture and Food Innovation, The University of Queensland, Brisbane, QLD 4072, Australia

**Keywords:** starch composites, zeaxanthin, encapsulation evaluation, storage stability, half-life time, in vitro digestion, release behavior

## Abstract

Zeaxanthin is a natural xanthophyll carotenoid and the main macular pigment that protects the macula from light-initiated oxidative damage, but it has poor stability and low bioavailability. Absorption of this active ingredient into starch granules as a carrier can be used to improve both zeaxanthin stability and controlled release. Optimization using three variables judged important for optimizing the system (reaction temperature of 65 °C, starch concentration of 6%, and reaction time of 2 h) was conducted for incorporation of zeaxanthin into corn starch granules, aiming for high zeaxanthin content (2.47 mg/g) and high encapsulation efficiency (74%). Polarized-light microscopy, X-ray diffraction, differential scanning calorimetry, and Fourier transform infrared spectroscopy showed that the process partially gelatinized corn starch; additionally, it showed the presence of corn starch/zeaxanthin composites, with the zeaxanthin successfully trapped in corn starch granules. The half-life time of zeaxanthin in corn starch/zeaxanthin composites increased to 43 days as compared with that of zeaxanthin alone (13 days). The composites show a rapid increase in zeaxanthin release with in vitro intestinal digestion, which is favorable for possible use in living systems. These findings could have application in designing effective starch-based carriers of this bioactive ingredient with enhanced storage stability and improved intestines-targeted controlled-release delivery.

## 1. Introduction

Zeaxanthin (β,β-carotene-3,3′-diol) is a xanthophyll carotenoid found in egg yolks and in very small amounts in dark-green leafy vegetables such as spinach; along with lutein, it is the principal carotenoid in the lens and macular region of the retina [1]. Epidemiological studies have demonstrated that zeaxanthin can play a protective role in certain eye diseases, such as age-related macular degeneration, cataracts, and retinitis pigmentosa, and can help reduce the risk of certain types of cancer, particularly of the breast and lung [2]. Despite its important physiological functions, the presence of conjugated double bonds in zeaxanthin means that it has a high tendency to lipid oxidation, which limits its stability during processing, storage, and cooking [3]. In addition, both the absorption efficiency and bioavailability of zeaxanthin are low, mainly due to its hydrophobic nature, which makes it slow to diffuse into aqueous digesta and to reach the intestinal cell epithelium for subsequent absorption in the digestive tract [4]. Developing new or improved techniques to improve zeaxanthin stability and bioavailability is therefore useful.

Due to its biodegradability and biocompatibility, starch is an excellent material for encapsulation of bioactive compounds in the food and biomedical fields [5]. Comparative studies have shown that starch-based systems give a higher encapsulation efficiency and provided better protection of food ingredients (e.g., flaxseed oil and flavors) against unfavorable environmental conditions than systems based on protein or gum Arabic [6,7]. There also has been increasing interest in using starch-based delivery systems to encapsulate polyphenols and control their release in the gastro-intestinal tract [8].

Various starch systems have been developed for encapsulation, including native starch granules, microporous starch, starch nanoparticles, substituted starch, cross-linked starch, hydrolyzed starch, amylose inclusion complexes, and other forms of modified starch [5]. Among them, unmodified native starch granules have the advantage of simplicity, being environmentally friendly, being labeled clean, completely non-toxic, and biocompatible. On the other hand, their applications and utility are currently much fewer than those of modified starch products, arising, among other reasons, from the hydrophilic nature of starch. Because of the presence of surface pores as well as channels in the granules in native starch, corn starch can encapsulate many active ingredients, including catechin, gallic acid, and caffeine, through infusion and binding [9]. For example, drop-wise addition of ethanol containing β-carotene to starch paste forms a starch/β-carotene composite, wherein both non-specific binding and also formation of ordered structures with starch chains enhance the stability of β-carotene against chemical oxidation and facilitate delivery of β-carotene to the colon [10]. Additionally, octenyl-succinylated high-amylopectin starches with a higher degree of branching and more rigid structure show the best colloidal stability, thus providing improved protection of β-carotene from degradation through hindrance of degradative enzymes or substances such as stomach acid [11]. 

Zeaxanthin has been encapsulated into *Opuntia monacantha* mucilage against the effects of light, heat, and oxygen, resulting in improved zeaxanthin retention during 28 days of storage [12]. The enhanced thermal stability of zeaxanthin and improved controlled release in simulated intestinal fluid was achieved by complex coacervation between gelatin and carboxymethyl cellulose (CMC), which is driven by electrostatic attraction between oppositely charged biopolymers [13]. However, there is no investigation on encapsulating zeaxanthin into starch granules, which would be a simple approach to protect zeaxanthin for controlled release. Incubation with corn-starch granules at various reaction temperatures (55–70 °C) had been shown to encapsulate lutein, resulting in improved storage stability compared with free lutein (~76% vs. ~15% in the retention index after 21 days), and targeted controlled release associated with starch hydrolysis in a simulated intestinal incubation of lutein [14].

Although the effect of reaction temperature on the encapsulation of lutein into starch granules has been reported, there is as yet no detailed investigation on how other necessary reaction parameters (such as starch concentration and reaction time) affect the formation and structural characterization of corn starch/zeaxanthin composites. Such an investigation could lead to improved functional properties (storage stability and release behaviors). Therefore, in this study, the effects of reaction temperature (50–70 °C), starch concentration (2–10%), and reaction time (0.5–4 h) on the zeaxanthin content and encapsulation efficiency of corn starch/zeaxanthin composites were explored. The corn starch/zeaxanthin composites so obtained were characterized by light microscopy (LM), polarizing microscopy (PM), scanning electron microscopy (SEM), X-ray diffraction (XRD), Fourier transform infrared spectroscopy (FTIR), small angle X-ray scattering (SAXS) and differential scanning calorimetry (DSC), and their functional properties (storage stability and release behaviors) were examined. This study can be used to indicate how conditions can be optimized in synthesizing composites using native starch granules to deliver sensitive bioactive ingredients as functional food supplements.

## 2. Materials and Methods

### 2.1. Materials

Native corn starch granules (amylose content ~28%) were kindly provided by Starpro Starch Co., Ltd. (Hangzhou, China). Zeaxanthin (purity ≥ 85%) was purchased from Macklin Biochemical Co., Ltd. (Shanghai, China). Pancreatin from porcine pancreas (Cat. No. P7545, activity 8 USP) and amyloglucosidase (Cat. No. A7095, activity 300 unit/mL) were purchased from Sigma-Aldrich Pty. Ltd. (Castle Hill, Australia). All other chemicals were of reagent grade and used as received.

### 2.2. Preparation of Corn Starch/Zeaxanthin Composites

Corn starch/zeaxanthin composites were prepared following a method described elsewhere [10,14] with minor modifications. Corn starch dispersions (100 mL; 2%, 4%, 6%, 8%, and 10%, *w*/*v*, dry basis) were prepared in deionized water, then heated in a water bath at various temperatures (50 °C, 55 °C, 60 °C, 65 °C, and 70 °C) under constant gentle stirring for 1 h. Ethanol (100 mL) containing 20 mg zeaxanthin was added dropwise to the resulting starch dispersions over a 30 min period under constant stirring. After incubation at various temperatures and times (0.5 h, 1 h, 2 h, 3 h and 4 h), the mixture was slowly cooled to room temperature. The resulting suspension was centrifuged at 3000× *g* for 20 min, and the precipitate was washed three times with ethanol solution (50%, *v*/*v*) to remove unencapsulated zeaxanthin. The corn starch/zeaxanthin composites so obtained were oven-dried at 40 °C for 24 h and passed through a 200-μm nylon sieve for further use.

### 2.3. Zeaxanthin Content and Encapsulation Efficiency

Each sample of the corn starch/zeaxanthin composites (10 mg, dry basis) was completely dispersed in 3 mL of dimethyl sulfoxide (DMSO) by vigorous vortexing. The resulting mixture was centrifuged at 3000× *g* for 10 min, and the zeaxanthin in the supernatant was analyzed from its absorbance at 458 nm [10,14]. The zeaxanthin content was determined as the amount of zeaxanthin contained within a given composite (mg zeaxanthin/g composite) as follows:

$$\mathrm{Zeaxanthin}\;\mathrm{content}\;(\mathrm{mg}/g)=\frac{\mathrm{Amount}\;\mathrm{of}\;\mathrm{zeaxanthin}\;\mathrm{within}\;a\;\mathrm{given}\;\mathrm{composite}}{\mathrm{Amount}\;\mathrm{of}\;a\;\mathrm{given}\;\mathrm{composite}}$$

The encapsulation efficiency is reported as the percent of zeaxanthin recovered from a composite in relation to the total zeaxanthin added during its formation as follows:

$$\mathrm{Encapsulation}\;\mathrm{efficiency}\;(\%)=\frac{\mathrm{Amount}\;\mathrm{of}\;\mathrm{zeaxanthin}\;\mathrm{recovered}\;\mathrm{from}\;a\;\mathrm{composite}}{\mathrm{Amount}\;\mathrm{of}\;\mathrm{the}\;\mathrm{total}\;\mathrm{zeaxanthin}\;\mathrm{added}\;}$$

### 2.4. Structural Characterization of Corn Starch/Zeaxanthin Composites

#### 2.4.1. Light and Polarized-Light Microscopy

A 1% (*w*/*v*, dry basis) sample suspension in the aqueous glycerol solution (1:1 water/glycerol) was characterized using an Olympus BX53 (Tokyo, Japan) with brightfield and polarized light [15].

#### 2.4.2. Scanning Electron Microscopy

For scanning electron microscopy (SEM), samples were mounted on aluminium stubs with double-sided sticky tape and sputter-coated with a thin film of gold using a Hitachi S-4800Ⅱ scanning electron microscope (Tokyo, Japan) operating at 10 kV accelerating voltage and 4 mm working distance with 500× magnification [16].

#### 2.4.3. X-ray Diffraction

The XRD analysis of samples was carried out using an X-ray powder diffractometer (D8, Bruker, Germany), operated at 40 kV and 40 mA. Samples were manually packed tightly onto the glass sample holder, and data collected over an angular range from 2θ = 4° to 35° with a step of 0.02° [14]. Their relative crystallinity was calculated as the ratio of the corresponding crystalline peak area to the total diffraction area, wherein the crystalline peak area and amorphous area were separated by PeakFit 4.0 software (Systat Software Inc., San Jose, CA, USA) following a published method [17].

$$\mathrm{Relative}\;\mathrm{crystallinity}\;=\frac{\mathrm{Crystalline}\;\mathrm{peak}\;\mathrm{area}\;}{\mathrm{Crystalline}\;\mathrm{peak}\;\mathrm{area}+\mathrm{amorphous}\;\mathrm{area}}$$

#### 2.4.4. Fourier Transform Infrared Spectroscopy

Samples were ground with KBr (1:150, *w*/*w*) manually in an agate mortar for 1 min, and then the above mixed powders (0.2 g) were pressed into pellets of 13 mm diameter and 1 mm thickness under 10 MPa using an automatic tablet press for analysis [18]. The FTIR spectra of samples were recorded over 400–4000 cm^−1^ using an Antains II FTIR spectrophotometer (Thermo Fisher Scientific, Inc., Waltham, MA, USA) [16].

#### 2.4.5. Small Angle X-ray Scattering

Samples were mixed with deionized water to achieve ~60% of total moisture content and equilibrated at ambient temperature (~25 °C) for 24 h. A small-angle X-ray scattering diffractometer (NanoSTAR, Bruker AXS Inc., Madison, WI, USA), equipped with a Vantec 2000 detector (Bruker, Berlin, Germany) and pinhole collimation for point focus geometry, were used for sample lamellar structure analysis, as described elsewhere [19]. The average repeat distance (i.e., thickness of the semicrystalline lamellae) of the amorphous and crystalline lamellar of each sample was calculated as *d* = 2π/*q*, where *d* (nm) is the lamellar repeat distance and *q* (nm^−1^) is the scattering vector, with *q* = (4π sinθ)/λ, where λ (nm) is the X-ray wavelength and 2θ is the scattering angle.

#### 2.4.6. Differential Scanning Calorimetry

Samples (3 mg, dry basis) were mixed with distilled water to achieve 70% total moisture content and hermetically sealed in stainless steel pans for 12 h to equilibrate. The thermal properties of samples were analyzed by differential scanning calorimetry (DSC-8000, Perkin Elmer, Waltham, MA, USA) with a scanning range from 30 °C to 150 °C at a rate of 10 °C/min [14].

### 2.5. Storage Stability

Residual zeaxanthin content (mg/g) in the samples was analyzed over 21 days of storage, and the zeaxanthin retention (%, the ratio of residual zeaxanthin content to initial zeaxanthin content) was plotted against storage time on a semi-log plot. The data are interpreted assuming simple first-order loss. The rate coefficient (*k*) and half-life time (*t*_1/2_) for the retention of zeaxanthin were calculated from these plots as ln (zeaxanthin retention) = *a* − *kt* and *t*_1/2_ = ln 2/*k* [20].

### 2.6. In Vitro Gastric and Intestinal Digestion

The release kinetics of zeaxanthin in the corn starch/zeaxanthin composites under in vitro simulated stomach and intestinal conditions was obtained with a modified method described elsewhere [10,14]. Samples (1 g, dry basis) were mixed with 20 mL of simulated gastric fluid containing pepsin (pH 1.2) to start the digestion progress and incubated at 37 °C with a stirring rate of 30 rpm for 20, 40, 60, 80, 100, and 120 min. Then, the above gastric chymes were combined with 20 mL of simulated intestinal fluid containing pancreatin and amyloglucosidase (pH 7.0) to start the simulated small-intestine digestion. The aliquots of gastrointestinal digestion were taken at different time points. The activities of pepsin, pancreatin, and amyloglucosidase were stopped by adding absolute ethanol, and digested aliquots were centrifuged at 4000× *g* for 5 min to separate undigested residues. The centrifuged aliquots were analyzed for zeaxanthin content as described in Section 2.3, and the zeaxanthin release (%) is reported as the percent release of zeaxanthin to the total zeaxanthin in the composites as follows:

$$\mathrm{Zeaxanthin}\;\mathrm{release}\;(\%)=\frac{\mathrm{Amount}\;\mathrm{of}\;\mathrm{zeaxanthin}\;\mathrm{release}\;}{\mathrm{Amount}\;\mathrm{of}\;\mathrm{the}\;\mathrm{total}\;\mathrm{zeaxanthin}\;\mathrm{in}\;\mathrm{the}\;\mathrm{composites}}$$

### 2.7. Statistical Analysis

All experiments were performed at least in duplicate unless otherwise specified, and statistical analysis was conducted using SPSS software (version 19.0, SPSS Inc., Chicago, IL, USA). Significant differences in the data were analyzed by analysis of variance (ANOVA) with Duncan’s multiple range test at the *p* < 0.05 confidence level.

## 3. Results and Discussion

### 3.1. Effect of Reaction Parameters on the Zeaxanthin Content and Encapsulation Efficiency

#### 3.1.1. Effect of Reaction Temperature

**Figure 1:**
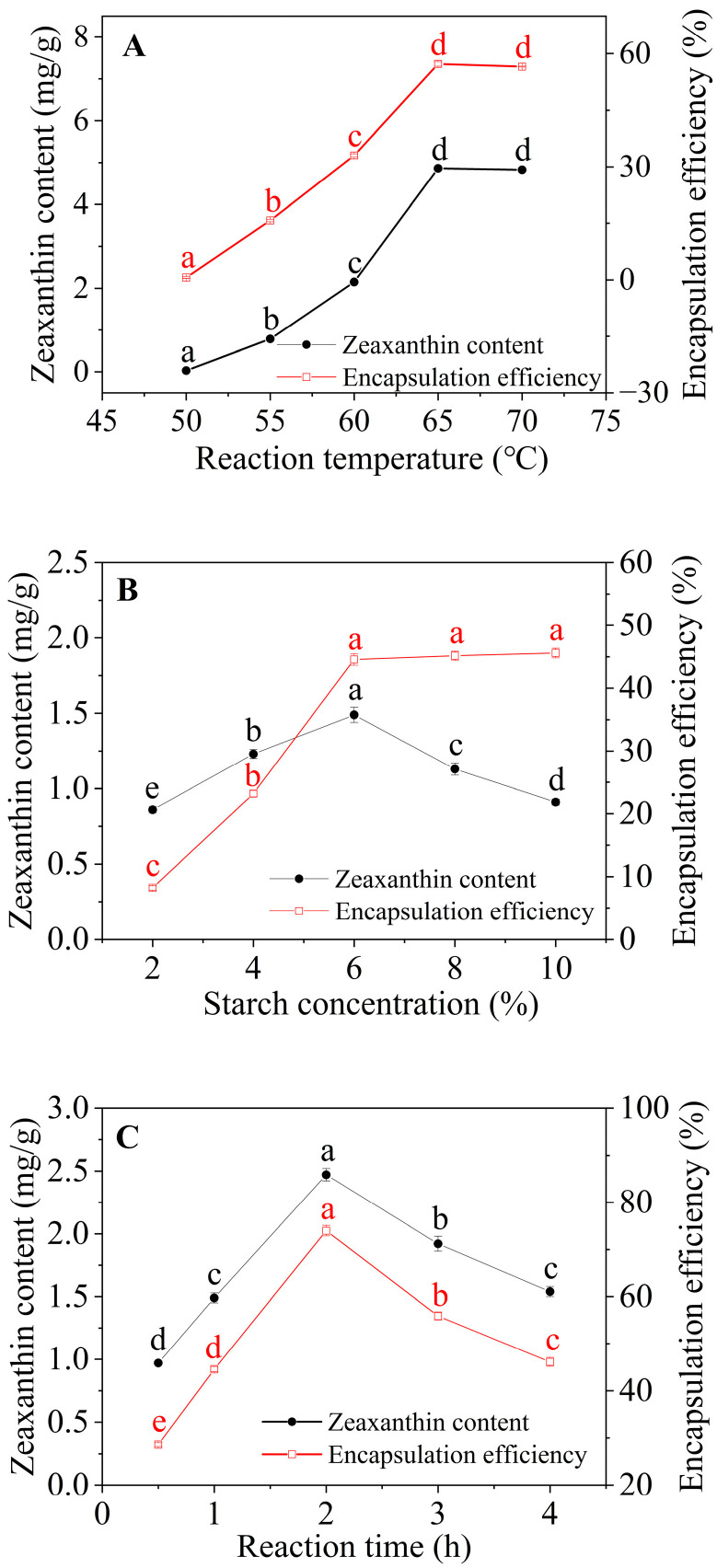
Zeaxanthin content and encapsulation efficiency of corn starch/zeaxanthin composites as functions of reaction temperature (**A**), starch concentration (**B**), and reaction time (**C**). Values with same letter in a row do not differ significantly (*p* > 0.05).

Figure 1A presents the zeaxanthin content and encapsulation efficiency of the corn starch/zeaxanthin composites as a function of reaction temperature (50–70 °C) for a starch concentration of 4% and reaction time of 1 h. Over the range of reaction temperatures from 50 to 65 °C, the zeaxanthin content and encapsulation efficiency of the composites increased from 0.03 to 4.86 mg/g and from 0.61% to 57.28%, respectively. This is attributed to an increase in sub-gelatinization temperature causing the swelling of starch granules and amylose leaching, both of which facilitate the entrapment of zeaxanthin molecules [21]. There was no significant difference in zeaxanthin content and encapsulation efficiency in the composites when the reaction temperature was increased from 65 °C to 70 °C, which can be explained in terms of the possible oxidation degradation of zeaxanthin because of the relatively high temperature [22]. Similar results have been observed for the encapsulation of lauric acid and of lutein into swollen corn-starch granules [14,23]. A reaction temperature of 65 °C was selected for subsequent runs.

#### 3.1.2. Effect of Starch Concentration

Figure 1B exhibits the zeaxanthin content and encapsulation efficiency of the corn starch/zeaxanthin composites as a function of starch concentration (2–10%) with a fixed reaction temperature of 65 °C and reaction time of 1 h. As starch concentration increased from 2% to 6%, zeaxanthin content and encapsulation efficiency of the corn starch/zeaxanthin composites increased significantly from 0.86 mg/g to 1.49 mg/g and from 8.23% to 44.56%, respectively. With starch concentration further increased from 6% to 10%, one sees a significant decrease to 0.91 mg/g in zeaxanthin content and no significant change in encapsulation efficiency. The results are ascribed to insufficient amounts of zeaxanthin (20 mg) with starch concentration above 6%. This hypothesis is supported by what happens with a further increase in starch concentration from 6% to 10%: a significant decrease from 1.49 mg/g to 0.91 mg/g in zeaxanthin content and no significant change for encapsulation efficiency. A similar result has been found for debranched starch/phosphatidylcholine inclusion complexes [24]. A starch concentration of 6% was therefore used for subsequent experiments.

#### 3.1.3. Effect of Reaction Time

Figure 1C shows the zeaxanthin content and encapsulation efficiency of the corn starch/zeaxanthin composites as a function of reaction time (0.5–4 h) at a reaction temperature of 65 °C and 6% starch concentration. As the reaction time increased from 0.5 h to 2 h, zeaxanthin content and encapsulation efficiency of the corn starch/zeaxanthin composites increased significantly from 0.97 to 2.47 mg/g and 28.60% to 74.00%, respectively, probably because the long reaction time enabled more zeaxanthin to access corn starch granules. On increasing the reaction time from 2 h to 4 h, zeaxanthin content and encapsulation efficiency decreased significantly. This might be because reacting at 65 °C for more than 2 h could cause slow complexation of corn starch granules with zeaxanthin at the fixed starch/zeaxanthin ratio resulting from the insufficient starch-based encapsulation matrix after achieving a critical value [24]. Another possible reason is that the low solubility of zeaxanthin in water limited its diffusion into the polymer phase especially with corn starch maintained in the non-solidified (semi-solid) state [5]. Thus, optimal conditions for zeaxanthin content (2.47 mg/g) and encapsulation efficiency (74%) in corn starch/zeaxanthin composites were: reaction temperature of 65 °C, starch concentration of 6%, and reaction time of 2 h to achieve the maximum values; these conditions were chosen for subsequent structural characterization and functional evaluation.

### 3.2. Structural Characterization

#### 3.2.1. Morphology

**Figure 2:**
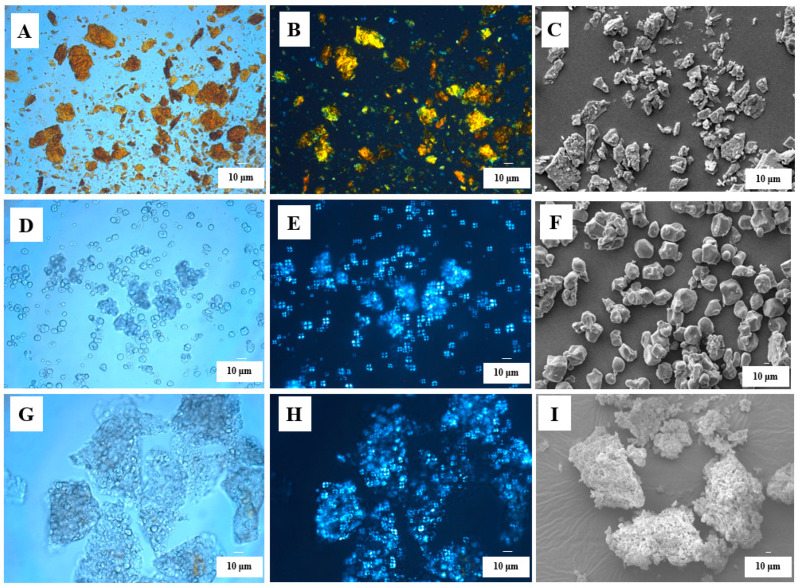
Zeaxanthin (Visible light) (**A**), Zeaxanthin (polarized light) (**B**), Zeaxanthin (scanning electron microscope) (**C**), corn starch (Visible light) (**D**), corn starch (polarized light) (**E**), corn starch (scanning electron microscope) (**F**), corn starch/zeaxanthin composites (Visible light) (**G**), corn starch/zeaxanthin composites (polarized light) (**H**), corn starch/zeaxanthin composites (scanning electron microscope) (**I**).

Figure 2 shows the images of zeaxanthin, corn starch, and corn starch/zeaxanthin composites using light, polarizing, and scanning electron microscopy. Under polarizing light, zeaxanthin showed a bright pattern with scattered distribution (Figure 2B), consistent with that under normal light (Figure 2A). Corn starch was polygonal shaped (Figure 2D) under normal light as reported previously [25], and its polarized-light micrograph exhibited a characteristic birefringence pattern with a Maltese cross centered at the hilum (Figure 2E), indicating a partially ordered arrangement of starch molecules. After reaction with zeaxanthin, large composite particles consisting of aggregated corn starch granules with weakened Maltese crosses were observed, suggesting the chosen reaction parameters did not fully gelatinize the corn starch granules. The surface of the corn starch granules appeared to be smooth with no distinct features (Figure 2D,F), and the corn starch/zeaxanthin composites displayed a rough appearance with thin lamellar structures (Figure 2G,I). This phenomenon is probably due to starch retrogradation after insufficient gelatinization, a complex process involving granule swelling, molecular rearrangement, and association [26]. A similar result was observed for ternary blends containing swollen maize starch, maize oil, and zein protein [25].

#### 3.2.2. Crystalline Characteristics

**Figure 3:**
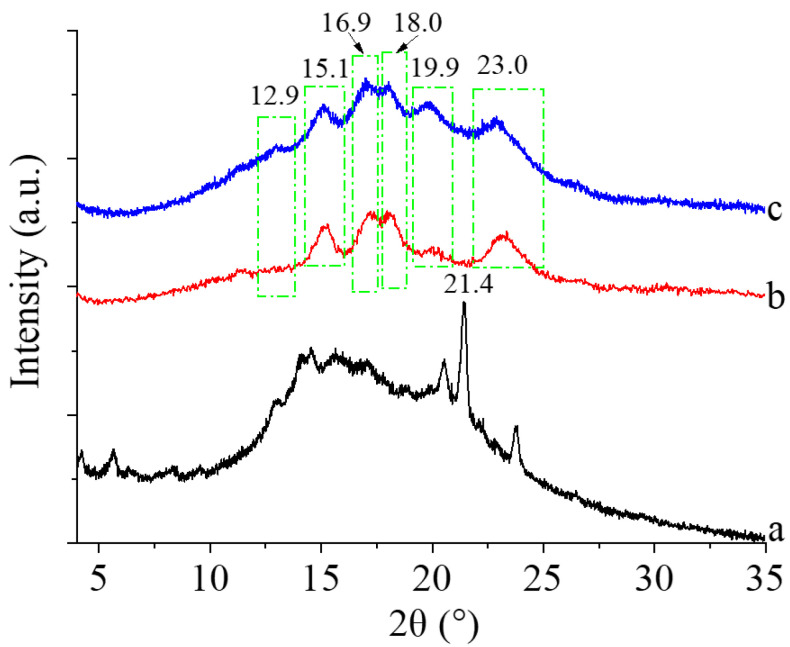
XRD diffraction patterns of zeaxanthin (a), corn starch (b), and corn starch/zeaxanthin composites (c).

XRD diffraction patterns of zeaxanthin, corn starch, and corn starch/zeaxanthin composites are shown in Figure 3. The diffractogram of zeaxanthin exhibited a large sharp peak at around 21.4° (Figure 3 (a)), indicating the crystallization of the zeaxanthin. A typical A-type crystalline structure with main diffraction peaks at 15.1°, 16.9°, 18.0°, and 23.0° (Figure 3 (b)) was observed for corn starch, in agreement with a previous report [23]. The diffractogram of the corn starch/zeaxanthin composites was slightly sharper than that of corn starch, especially the peaks at 12.9° and 19.9° (Figure 3 (c)), which is a typical diffraction pattern of V-type amylose inclusion complexes. This indicates the formation of leached amylose/zeaxanthin complexes, similar to previous studies of swollen cornstarch/lutein composites and corn-starch-β-carotene composites [10,14].

#### 3.2.3. FTIR

**Figure 4:**
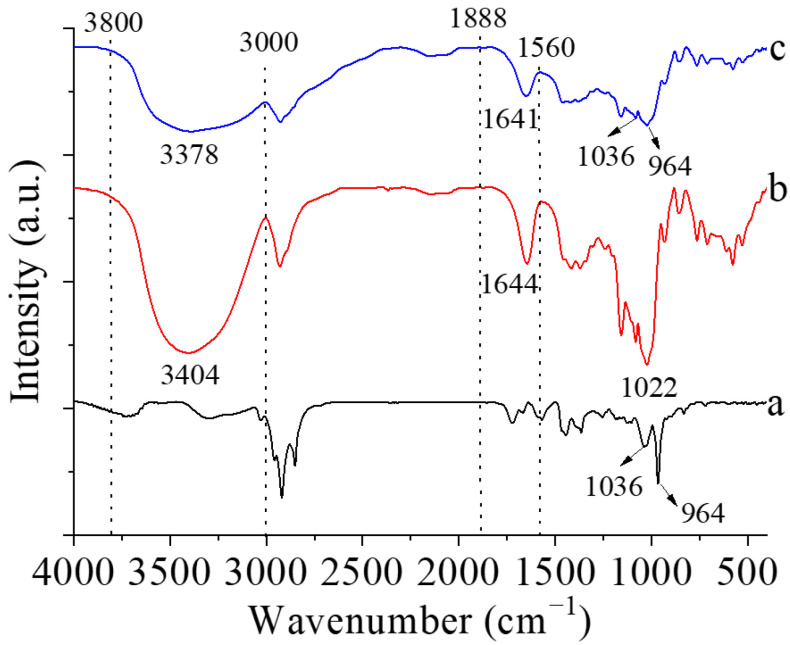
FT-IR spectra of zeaxanthin (a), corn starch (b), and corn starch/zeaxanthin composites (c).

Figure 4 shows the FTIR spectra of zeaxanthin, corn starch, and corn starch/zeaxanthin composites. For zeaxanthin, the very sharp band at 1036 cm^−1^ and the peak at 964 cm^−1^ (Figure 4 (a)) correspond to the conjugated C=C stretching vibration, respectively. Corn starch exhibited the main adsorption bands at 3404, 1644, and 1022 cm^−1^, which were related with the O–H stretching, water adsorption, and stretching of the glucose ring, respectively [27]. Compared with the spectrum of corn starch (Figure 4 (b)), two new peaks at 1036 cm^−1^ and 964 cm^−1^ were observed for the corn starch/zeaxanthin composites (Figure 4 (c)), confirming zeaxanthin was successfully entrapped into the corn starch granules. In addition, the peaks of the composites at 3000 to 3800 cm^−1^ and 1560 to 1888 cm^−1^ became wider and red-shifted with the addition of zeaxanthin, implying the presence of intermolecular hydrogen bonds between zeaxanthin and the amorphous region of the starch molecule [28].

#### 3.2.4. Cluster and Fractal Characteristics

**Figure 5:**
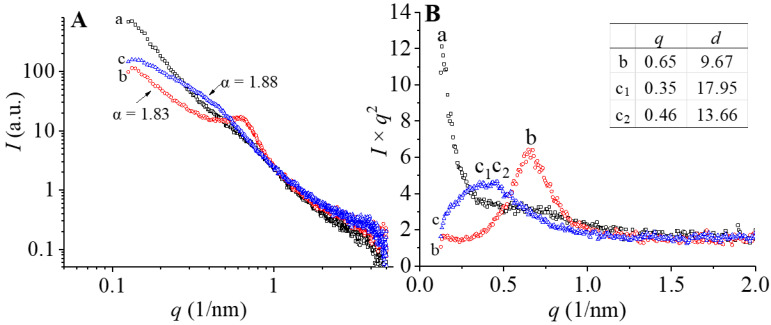
Log-log (**A**) and *I* × *q*^2^ (**B**) SAXS data of zeaxanthin (a), corn starch (b), and corn starch/zeaxanthin composites (c). (*I*: intensity; α: the exponent in the scattering power-law equation: *I*~*q*^−α^; *D*: fractal dimension; *d*: the lamellar repeat distance; *q*: the scattering vector).

Characterization of the cluster and fractal structure of zeaxanthin, corn starch, and corn starch/zeaxanthin composites was performed using SAXS, and their scattering patterns are presented in Figure 5A,B. The fractal structures can be characterized by the fractal dimension *D*, which is related to the scattering power-law equation *I*~*q*^−α^, where *I* is the SAXS intensity and α is an exponent, which can be used to calculate the value of *D* of the surface/mass fractal structure [29]. The value of α can be obtained from the slope of a log-log SAXS plot. When 3 < α < 4, the scattering objects can be seen as being a surface fractal structure, with the fractal dimension *D*_s_ = 6 − α. When 1 < α < 3, the scattering objects can be seen as being a mass fractal structure, with the fractal dimension *D*_m_ = α. *D*_s_ can be seen as an indicator of the degree of smoothness, with *D*_s_ = 2 when the surface of the scattering objects is smooth. *D*_m_ indicates the compactness, and the *D*_m_ values of a linear arrangement, a surface-like arrangement, and a regular arrangement such as a cube or sphere, being 1, 2, and 3, respectively [30]. The scattering exponents of corn starch and the corn starch/zeaxanthin composites were 1.83 and 1.88, indicating both had a fractal structure with *D*_m_ = 1.83 and 1.88 (Figure 5A (b,c)), respectively. The *D*_m_ value of the corn starch/zeaxanthin composites was slightly higher than that of corn starch, implying tighter molecular arrangement in the corn starch/zeaxanthin composites, inferred by analogy to a previous study of debranched starch/phosphatidylcholine inclusion complexes [24].

The *I* × *q*^2^ SAXS patterns of zeaxanthin, corn starch, and corn starch/zeaxanthin composites after Lorentz correction [31] is presented in Figure 5B. Here, *d* = 2π/*q*, where Bragg distance *d* (nm) is the lamellar repeat distance (i.e., thickness of semicrystalline lamellae) and *q* (nm^−1^) is the scattering vector. Corn starch shows a characteristic peak at about *q* = 0.65 nm^−1^ (Figure 5B (b)), which corresponds to a *d* of 9.67 nm. This peak originates from the semicrystalline structure (i.e., alternating crystalline and amorphous structure) of corn starch granules, consistent with the literature [29]. Two new shoulder peaks (peak 1 and peak2 marked in c_1_ and c_2_) were observed at *q* = 0.35 nm^−1^ and 0.46 nm^−1^ for the corn starch/zeaxanthin composites (Figure 5B (c_1_ and c_2_)), corresponding to two semicrystalline structures with *d* of 17.95 nm and 13.66 nm in the composites, respectively. The first peak (*q* = 0.35 nm^−1^) in the corn starch/zeaxanthin composites should be closely related to the formation of amylose/zeaxanthin V-type complexes [24], and the second peak (*q* = 0.46 nm^−1^) is attributed to starch retrogradation after insufficient gelatinization, resulting in less disordering occurring after the encapsulation [26].

#### 3.2.5. Thermal Properties

The thermal properties of zeaxanthin, corn starch, and corn starch/zeaxanthin composites, namely the onset temperature (*T*_o_), peak temperature (*T*_p_), conclusion temperature (*T*_c_), and enthalpy (Δ*H*), are presented in Table 1. Corn starch exhibited a broad unimodal endotherm ranging from 76.1 to 85.7 °C, higher than those of previous studies (64.1 to 74.5 °C [14] and 69.7 to 77.8 °C [32]), which is probably related to the internal crystalline structure of starch and its heat stability. The corn starch/zeaxanthin composites showed a higher *T*_o_, *T*_p_, *T*_c_, and Δ*H* compared with corn starch, indicating more higher crystallinity, consistent with the SAXS data. A similar observation was reported in the starch/zein nanocomposites, which exhibited a thermogram shifted to higher temperatures due to molecular interactions between the starch chain and zein [16]. A second endothermic transition above 100 °C was observed in the composites, corresponding to V-type amylose inclusion complexes [10,14], further confirming the formation of amylose/zeaxanthin V-type complexes, consistent with XRD data.

### 3.3. Storage Stability

**Figure 6:**
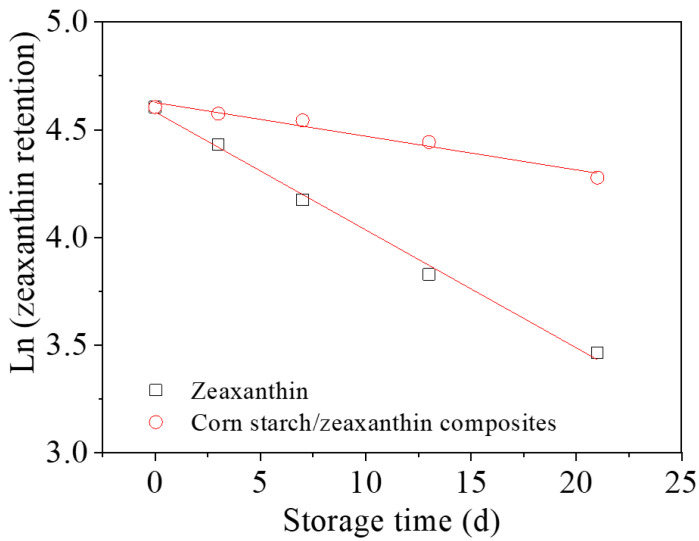
Zeaxanthin retention from pure zeaxanthin and from corn starch/zeaxanthin composites over 21 days of storage time.

Figure 6 shows the zeaxanthin retention in the pure zeaxanthin and corn starch/zeaxanthin composites over 21 days of storage time. The zeaxanthin retention of zeaxanthin and the corn starch/zeaxanthin composites decreased to different extents as a function of storage time, due to the high degradation susceptibility of zeaxanthin when exposed to environmental factors such as oxygen and light [1,12]. The half-lives of zeaxanthin by itself and in the corn starch/zeaxanthin composites are given in Table 2. The *t*_1/2_ of zeaxanthin in the corn starch/zeaxanthin composites increased from 13 to 43 days after encapsulation with corn starch granules with optimized reaction parameters; after 21 days of storage, the zeaxanthin retention of the corn starch/zeaxanthin composites was 72.1% compared with that of zeaxanthin alone (32.0%). This compares with the retention of zeaxanthin in *Opuntia monacantha* mucilage-based nanoparticles and nanoemulsion of 46% and 30%, respectively, after 21 days of storage at 25 °C [12]. This shows that the encapsulation of zeaxanthin into corn starch granules protects the zeaxanthin from degradation.

### 3.4. In Vitro Digestion

**Figure 7:**
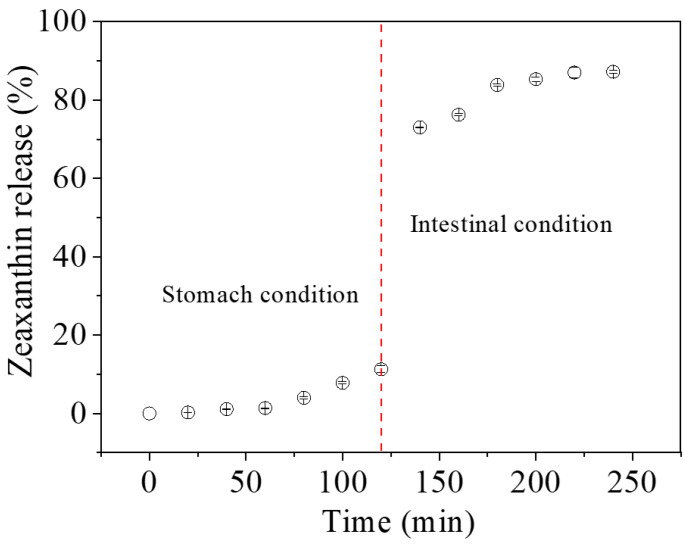
In vitro stomach and intestinal digestion of corn starch/zeaxanthin composites. The break at 120 min is the end point of stomach digestion and the starting point of intestinal digestion. All symbols in this figure are hollow circle with their corresponding error bars.

Figure 7 shows in vitro stomach and intestinal digestion of the corn starch/zeaxanthin composites. The low release rate of zeaxanthin in the corn starch/zeaxanthin composites is seen in the gastric stage, which was probably related to the absence of starch-digestion enzymes (i.e., starch was not hydrolyzed at this stage) and the structural disintegration of composite surface resulting from the low pH of simulated gastric fluid (containing gastric acid) [33]. This result was different from protein-based composites that would lose the structural integrity during the gastric digestion process due to the presence of pepsin [34]. In the simulated intestinal digestion, pancreatin containing α-amylase could hydrolyze starch molecules into linear glucans and branched dextrins by an endo mechanism at inner α-1,4 glucosidic linkages, and amyloglucosidase could further hydrolyze the degraded products from pancreatic α-amylase to absorbable glucose, both of which could lead to the degradation and structural destruction of starch granules [35]. A rapid increase in zeaxanthin release in corn starch/zeaxanthin composites occurred within the first 20 min of intestinal digestion, which may be related with the hydrolysis rate of starch-based encapsulation matrix referenced from a previous study on the swollen cornstarch/lutein composites [14]. Starch-based intestinal-targeted release behavior of bioactive compounds has also been implemented in corn starch/β-carotene composites [10] due to their similar complexing process and structural characteristics. The results suggest here that corn starch/zeaxanthin composites are suitable for intestinal-targeted delivery.

### 3.5. The Underlying Mechanism on the Formation of Corn Starch/Zeaxanthin Composites

**Figure 8:**
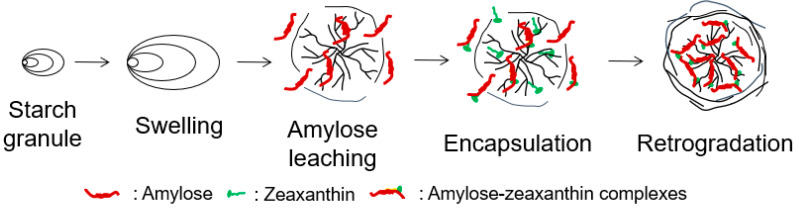
Schematic diagram of the formation of corn starch/zeaxanthin composites.

Figure 8 presents a schematic diagram of the formation of corn starch/zeaxanthin composites. Hydrothermal treatment at a sub-gelatinization temperature initiates granule swelling, and some amylose can leach out from the granules [21]. Corn starch gelatinization takes place when the hydrothermal treatment is set close to the onset temperature *T*_o_ (76.1 °C, Table 2). Corn starch/zeaxanthin composites are formed at the optimized reaction parameters (65 °C reaction temperature, 6% starch concentration, and 2 h reaction time). Figure 1 in this study could be associated with starch retrogradation after partial gelatinization, evidenced by light, polarized light, and electron microscope images (Figure 2, showing weakened Maltese crosses and a rough appearance with thin lamellar structures) and XRD patterns (Figure 3, typical of that for an A + V-type crystallinity). The FTIR spectra (Figure 4, two new peaks for zeaxanthin) are consistent with zeaxanthin being successfully trapped in the corn starch granules; the XRD patterns (Figure 3, showing a V-type crystalline pattern) and DSC data (Table 1, a second endothermic transition above 100 °C) also indicate the formation of V-type leached amylose/zeaxanthin complexes. SAXS patterns (Figure 5, two semicrystalline structures with *d* = 17.95 and 13.66 nm) show the presence of both amylose/zeaxanthin complexes and retrograded starch after insufficient gelatinization, all of which would be expected to contribute to enhanced storage stability (Figure 6 and Table 2) and intestinal-targeted delivery (Figure 7) of zeaxanthin in corn starch/zeaxanthin composites. The zeaxanthin residing within the core of the composites may have been protected by amorphous and/or ordered structures of retrograded starch after insufficient gelatinization, which could inhibit exposure of zeaxanthin to environmental factors such as oxygen and light, similar to what is seen in composites of corn starch and β-carotene [10]. The presence of roughness with thin lamellar structures and an enhanced V-type crystalline may substantially retard the accessibility of digestive enzymes to starch chains [36], thus enhancing the controlled delivery of intestinal-targeted corn starch/zeaxanthin composites, which could be used as functional food supplements.

## 4. Conclusions

This study investigated the formation, structures, and functional properties of composites between native corn starch granules and zeaxanthin, aiming for the encapsulation of zeaxanthin as an active ingredient in corn starch as an inert carrier. The optimal conditions achieved here were a zeaxanthin content of 2.47 mg/g and encapsulation efficiency of 74.00% in the corn starch/zeaxanthin composites, at a reaction temperature of 65 °C, 6% starch concentration, and 2 h reaction time. The resulting corn starch/zeaxanthin composites displayed a rough appearance with thin lamellar structures and enhanced V-type starch crystallinity. FTIR confirmed that zeaxanthin was successfully entrapped in the corn starch granules. XRD, SAXS, and DSC data together provided evidence that the corn starch/zeaxanthin composites consisted of amylose/zeaxanthin complexes and retrograded, but incompletely gelatinized, corn starch. Encapsulation of zeaxanthin into corn starch granules could protect zeaxanthin from storage degradation and achieve intestinal-targeted delivery. These could have applications in designing effective starch-based carriers of bioactive ingredients with enhanced storage stability for intestinal-targeted delivery, which would be beneficial for their potential use in the sensitive food supplement.

## Figures and Tables

**Table 1:** Thermal properties of zeaxanthin, corn starch, and corn starch/zeaxanthin composites.

	Zeaxanthin	Corn Starch	Corn Starch/Zeaxanthin Composites
Peak 1			
*T*_o_ (°C)	68.5 ± 1.2 ^b^	76.1 ± 1.0 ^a^	76.3 ± 1.1 ^a^
*T*_p_ (°C)	72.5 ± 0.5 ^b^	81.4 ± 0.6 ^a^	81.5 ± 0.6 ^a^
*T*_c_ (°C)	75.8 ± 0.2 ^b^	85.7 ± 0.9 ^a^	86.5 ± 1.1 ^a^
Δ*H* (J/g)	6.3 ± 0.6 ^a^	5.6 ± 0.9 ^b^	5.8 ± 1.3 ^b^
Peak 2			
*T*_o_ (°C)	-	-	100.8 ± 0.5
*T*_p_ (°C)	-	-	105.9 ± 0.6
*T*_c_ (°C)	-	-	109.0 ± 0.5
Δ*H* (J/g)	-	-	0.47 ± 0.05

Values with same letter in a row do not differ significantly (*p* > 0.05).

**Table 2:** Storage stability of zeaxanthin and corn starch/zeaxanthin composites over 21 days storage.

Samples	R^2^	*t*_1/2_ (d)	Retention after 21 d (%)
Zeaxanthin	0.99	13	32
Corn starch/zeaxanthin composites	0.96	43	72

## Data Availability

The datasets generated for this study are available on request to the corresponding author.

## Abbreviations

ANOVA, analysis of variance; CMC, carboxymethyl cellulose; DMSO, dimethyl sulfoxide; DSC, differential scanning calorimetry; FTIR, Fourier transform infrared spectroscopy; GOPOD, glucose oxidase/peroxidase reagent; LM, light microscopy; PM, polarizing microscopy; SAXS, small angle X-ray scattering; SEM, scanning electron microscopy; XRD, X-ray diffraction.
